# X-ray computed microtomography datasets for osteogenic nanofibrous coated titanium implants

**DOI:** 10.1038/s41597-022-01400-8

**Published:** 2022-06-18

**Authors:** Siddhartha Das, Kanchan Dholam, Sandeep Gurav, Kiran Bendale, Arvind Ingle, Bhabani Mohanty, Pradip Chaudhari, Jayesh R. Bellare

**Affiliations:** 1 Department of Biosciences and Bioengineering, Indian Institute of Technology Bombay, Mumbai, 400076 Maharashtra India; 2 Department of Chemical Engineering, Indian Institute of Technology Bombay, Mumbai, 400076 Maharashtra India; 3 Department of Dental and Prosthetic Surgery, Tata Memorial Centre, HBNI, Mumbai, 400 012 Maharashtra India; 4 Advanced Centre for Treatment, Research and Education in Cancer, Navi Mumbai, 410 210 Maharashtra India; 5 Homi Bhabha National Institute (HBNI), Training School Complex, Anushakti Nagar, Mumbai, 400085 India; 6 Wadhwani Research Centre for Bioengineering, Indian Institute of Technology Bombay, Mumbai, 400076 Maharashtra India

**Keywords:** Preclinical research, Three-dimensional imaging

## Abstract

Surface modifications of titanium implant influences the quality of osseointegration and are associated with favourable treatment prognosis in orthopaedic and cranio-maxillofacial cases. Hence, unlike previous works, the peri-implant region details of our novel osteogenic nanofibrous coated implants placed in rabbits (n = 6 + 1) were recorded over a 12-week period using a micro-CT imaging system. In this unique contribution, we have created a computed tomography (CT) library of rabbit’s tibiae anatomy with osteogenic nanofibrous coated/uncoated implants and are introductory useful assets for investigating the correlation between osteogenic nanofibers coated implants and its effect on improved osseointegration. Apart from using this CT dataset to conduct serial 2D image studies, three-dimensional (3D) reconstructions, assessing segmentation algorithms and developing adequate image quantitation tools, there may be positive applications of these in comparative investigations of similar or related preclinical as well as future clinical studies, further design planning, development etc. required for evolution of implants beyond the present state of art.

## Abstract (summary)

**Table:** 

Measurement(s)	Bone microstructure and quality
Technology Type(s)	High-resolution imaging technology
Factor Type(s)	Osseointegration
Sample Characteristic - Organism	Oryctolagus cuniculus
Sample Characteristic - Environment	Sterile Environment
Sample Characteristic - Location	India

## Background & Summary

Micro-computed tomography (micro-CT) with its space resolution, known to visualize and create cross-sectional bone microarchitecture through a range of computed attenuation-based parameters and further could be used to build a volume using tri-dimensional reconstruction technology^1^. Titanium implants, recognized for its lower linear attenuation coefficients^2^, advantageous for micro-CT investigations, on the other hand, has a broad range of application in medicine such as those used in maxillofacial surgery^3^, prosthetics^4^, spinal fusion cages^5^, etc. and a vast number of procedures towards improved implant osseointegration and peri-implant bone quality are thus being subjected to continuous technological research and innovations^6–8^. However, most of these information and available data sets are focused purely on *metallo-surface* modifications. Besides that, supposedly expected time for implant osseointegration in humans are rather time consuming and are believed to be at least in the range of 3-4 months^9^. Hence, in an effort to shed more light on current understanding on the dynamics of ideal implant surface with its time-efficient osseointegration in relation to our novel osteogenic nanofibrous coating, we performed an *ex vivo* x-ray micro-CT scan on tibiae obtained from 7 rabbits using a micro-CT imaging system known to gauge structure-function correlation for biomaterials in bones^10^.This easy and economical osteogenic nanofibrous coating on the titanium implant surface mimics extracellular matrix and includes both osteoinductive and osteoconductive chemicals resulting in differentiation of stem cells present in the peri-implant niche within the bone to osteoblast and thereby results in enhanced osseointegration of the implant^11,12^.These initiatory analysis of the aforementioned micro-CT datasets has been published in an associated journal^12^. However, we believe by sharing the contribution publicly we provide an additional opportunity to fellow researchers for managing and opine their own assessment in the performance, analysis or interpretation of the first and distinctive micro-CT datasets of peri-implant zone regenerated in response to our osteogenic nanofibrous coated implants. Previously, in the pilot animal trial^11^, and supplementary material^12^, to our earlier published articles, we have delineated the measurable parameters containing uniform volume of interest (VOI) between two subsequent stacks, selected adjacent to the implant surfaces for reliable density and morphology measurements that are necessary for quantification and comparison of regenerated peri-implant tissue. In the data descriptor, the introduced micro-CT data sets presents essential new morphological and functional information^13^ that can be utilized to gain insight and further understanding of implant-bone osseointegration and enhance regeneration at the interface. Therefore, the data assembled by us will be useful to a broad range of investigational, preclinical and clinical applications and also serve as a credible source of information that could be used as a starting point for further scrutinization regarding engineered tissues in response to the novel osteogenic nanofibrous coated implants.

## Methods

The details discussed in this paper are expanded versions of descriptions outlined in our earlier published articles^11,12^. In the study, the bio-response of osteogenic nanofibrous coated (test) and uncoated (control) titanium implants within the bone were investigated^11,12^. The test implants were coated with osteogenic nanofibres composed of composite blend of polycaprolactone 5.0% (w/v), gelatin type A 0.5% (w/v), dexamethasone 0.032% (w/v), β-glycerophosphate 0.5% (w/v), ascorbic acid 0.04% (w/v) and hydroxyapatite 0.04% (w/v) in 2,2,2-Trifluoroethanol.The titanium implants were coated with a modified electrospinning apparatus by placing the titanium screw (attached to the rotating shaft of a DC motor) in between the syringe tip and collector plate^11,12^.

### Study specimens

Six anatomically and physiologically healthy adult male New Zealand White rabbits (Oryctolagus cuniculus), one of the closest phylogenetic relatives to humans, in the range of 6-7 months old, weighing 2.89 ± 0.05 kgs were selected for the investigation after approvals from institutional animal ethics committee. Commercially available screw type titanium implants and its coated version were installed in the tibial diaphysis of those rabbits for the proof-of-concept trial. Control (uncoated) implants were placed in right tibia and test (coated) implant were placed in left tibia of same rabbit. Hence, a total of 4 implants (2 coated and 2 uncoated) were placed in each rabbit. Test and control implant were placed in the same rabbit in left and right tibia respectively to nullify the effects of inter-animal variability. Further, as the long bones of rabbit exhibited some inherent normal anatomical variations/individual structural differences and constrain us from using surgical guide, the predetermined sites in tibiae for implant placement were thus meticulously selected after detailed pre-operative radiographic evaluation leading to identification of sites that were roughly equivalent for all rabbit study models. All rabbits were acclimatized for two weeks in the disinfected environment maintained under standardized conditions i.e. 20 °C ± 5 °C temperature, 55% ± 5% humidity, 12-hour light-dark cycle and free access to autoclaved water and commercial diet. Ethical approval for the study was granted by the panel of ethics committee for laboratory animal research of ACTREC, Navi Mumbai WIHC/3596—TMH-IITB-ACTREC (Animal study proposal no. 03/2016) and was performed as per the guidelines of the Committee for the Purpose of Control and Supervision of Experiments on Animals (CPCSEA), Ministry of Social Justice and Empowerment, Government of India.

### Specimen preparation for micro-CT scanning

Peri-implant surgical wound healing was allowed for specified period for each rabbit as per study design^12^. i.e. till the end of 2^nd^, 4^th^, 6^th^, 8^th^ and 12^th^ week for the study models to gauge the quality of regenerated peri-implant tissue. Each animal was later sedated (ketamine, 35 mg kg^−1^ and xylazine, 5 mg kg^−1^) and was euthanized by intravenous administration of Thiopental 100 mg ml^−1^ as per their respective schedule. The fresh samples i.e., the tibia with test and control implants were obtained followed by careful excision of skin and fascia immediately, placed on ice, and were scanned within 12 hours using a micro-CT imaging system.

### Micro-CT scanner

**Fig. 1:**
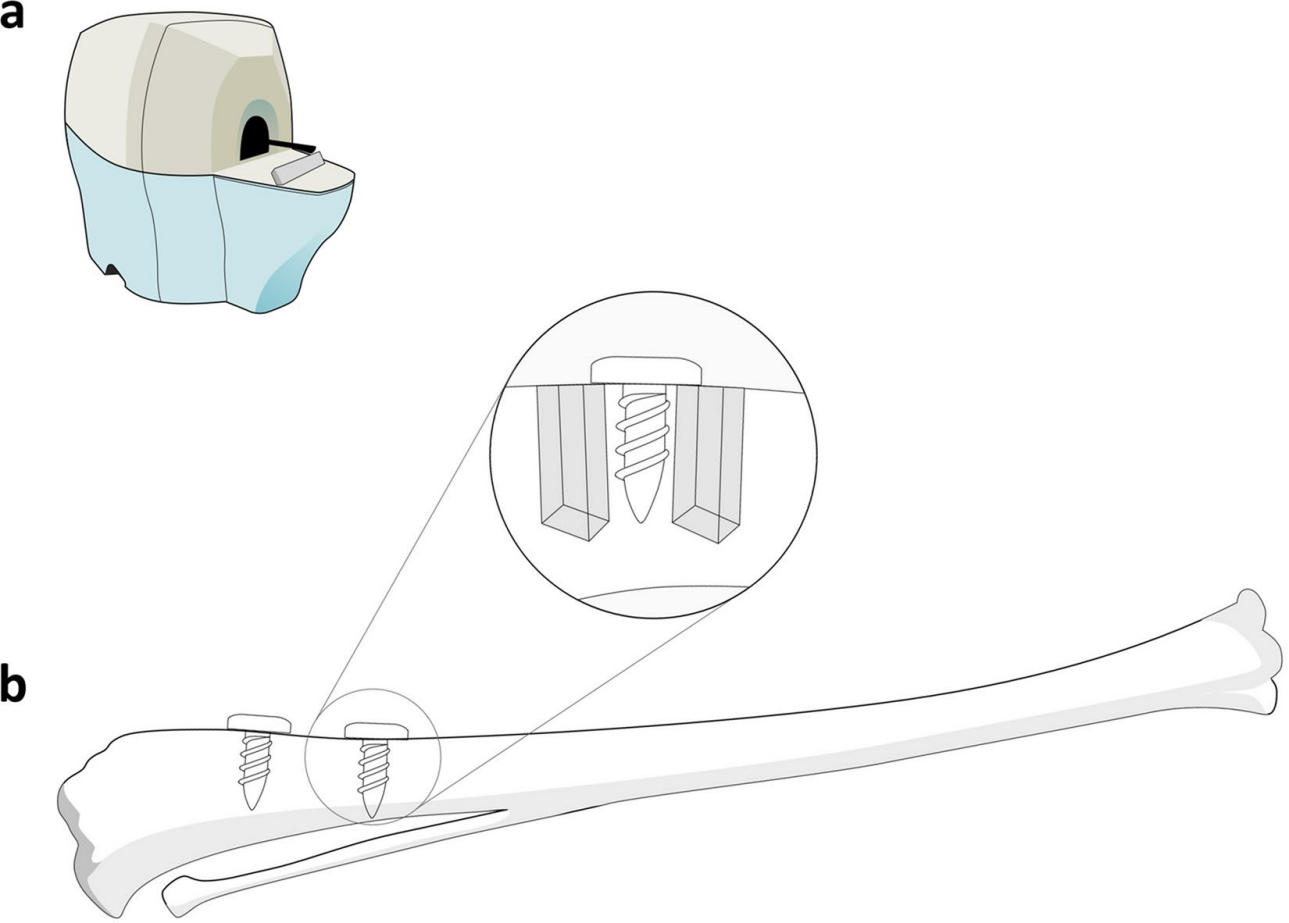
Assessment of peri-implant bone. (**a**) A preclinical micro-CT imaging system was used to evaluate the quality of peri implant bone (**b**) in the mesial and distal regions (inset) for implants placed nearer and far to proximal epiphysis.

The images were acquired using a micro-CT imaging system (Gamma Medica-Ideas, FLEX™ Triumph™ Pre-Clinical Imaging System, Northridge, CA, USA) (Fig. 1a) by scanning along the long axis of the specimen to compare the peri-implant areas (Fig. 1b). The detail operational parameters and specifications are presented in Table 1. Further, the focal spot used in the micro-CT system was 84 μm with 2X magnification having field of view (FOV) 59.2 mm^11,12^. The data was observed and analysed by GE MicroView CT program and OsiriX software respectively.

**Table 1:** Technical specifications and details of micro-CT imaging system used in the study.

Particulars	Type/Values	Description
X-ray source	Tungsten	The CT tube have tungsten as target material with cone angle adjusted to 38°
X-ray detector	CsI (Cesium Iodide) flipped scintillator plate	CMOS-based device with CsI flipped scintillator material with pixel array and 2240 × 2368 pixel matrix and 50 µm pitch.
Operating Voltage (kV)	90	Samples containing metal are scanned with high tube voltages to reduce artefacts
Beam Current (mA)	790	Samples containing metal are scanned with high tube current to reduce artefacts
Exposure Time (ms)	600	Lower exposure time is used to provide a suitable signal to noise ratio
Slice Spacing (mm)	1.2	Midpoint distance between the adjacent slices
Slice Thickness (mm)	0.225	A lower slice thickness results in higher image details and anatomical descriptions
Voxels size (µm)	5	Dimension of unit isotropic volumetric 3D pixel
Duty cycle	—	Continuous operation

### Data selection

Twelve micro-CT data sets of tibial implant bone complex (Fig. 1b) from six rabbits were obtained as DICOM files from the raw data generated in Small Animal Imaging Facility, Oncology Group, ACTREC, Navi Mumbai^14^. All the data sets were acquired immediately at the end of respective study period. Manufacturer’s instructions for quality control system calibration such as gantry calibration and camera calibrations were followed prior to data acquisition for achieving optimal acquisition quality in terms of parameters that affect the settings (includes x-ray voltage, spot size and binning mode etc.) After scanning, datasets with defined bone contours were inspected visually for possible presence and elimination (if any) of scanning artefacts. DICOM Data Transfer Tool and its protocol (http://microview.sourceforge.net) was used for obtaining DICOM files.

### Software versions

CT acquisition was performed with Triumph XO 4.1.1.0 © Gamma Medica – Ideas. Gantry and camera calibration performed before each study acquisition. After image acquisition MicroView (V2.1.2) 3D Image Viewer & Analysis Tool (http://microview.sourceforge.net) was used for 2D and 3D image viewing and analysis and CT Calibration plugin of MicroView was used to measure three different ROI’s within an image. The DICOM files exported to OsiriX® 8.5 software (Osirix Foundation, Geneva, Switzerland, https://www.osirix-viewer.com/) for 3D reconstruction and advanced computation.

### 3D segmentations and reconstruction

The DICOM files from the CT scans were imported into OsiriX^®^ 8.5 software (Osirix Foundation, Geneva, Switzerland, https://www.osirix-viewer.com/) for micro-CT imaging, segmentation, 3D-volume visualization, rendering and analysis. Numerous voxels i.e. 3D matrix of volumetric elements constitutes each micro-CT slice and represents average attenuation value, demonstrating the intensity numerical readings and are assigned as its respective CT number (grayscale). Various imaging parameters such as image acquisition, 3D reconstruction and post-image processing modes recognizes these variation in CT number and subsequently utilize them for quantification and evaluation of regenerated osseous tissue. The gray scale obtained by the micro-CT scanner were calibrated as per standard procedures of the manufacturer. Segmentation and 3D reconstruction were performed by three experienced evaluators for nullifying ambiguity. For segmentation purpose, manual outlining of defined anatomical contour was performed meticulously by all three evaluators individually for DICOM files obtained from all rabbit study models and were blinded for their own results and those of others. Regions with increased density and compactness, for instance, coated and uncoated titanium screws appears brighter, when compared to other biological structures in the slices.

**Fig. 2:**
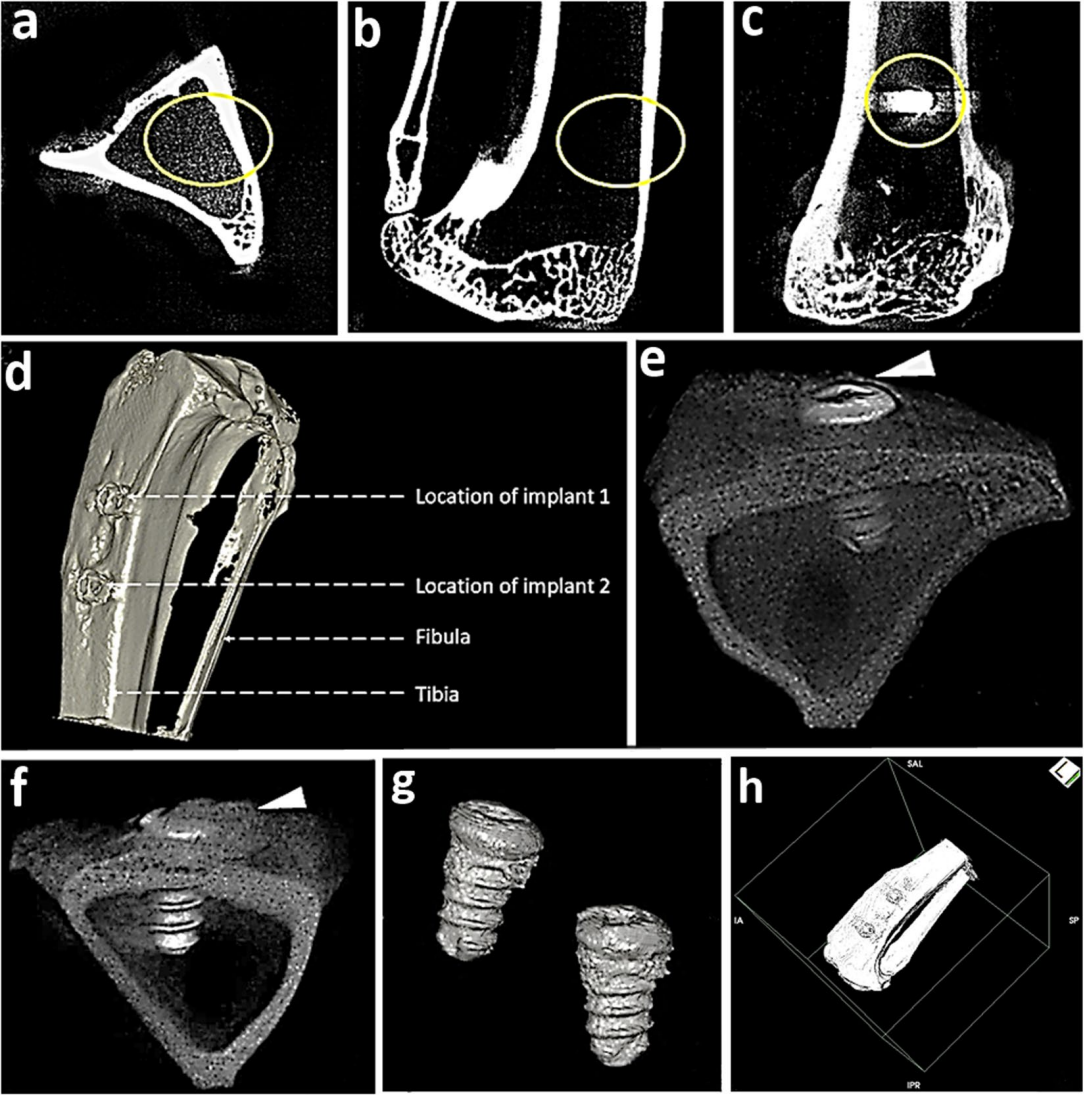
Assessment of 3D implant bone volume and tibial anatomy in rabbit, post surgery. (**a**–**c**) Planes for VOI calculations (~11.25 mm in axial and sagittal plane, ~16.20 mm in coronal plane, slice thickness is 0.225 mm) (**d**) high resolution anatomical 3D X-ray microtomography of right tibia specifying location of control implants (**e**) tibial-test implant bone complex (**f**) tibial-control implant bone complex (**g**) test implants in tibia after 12 weeks of healing (**h**) 3D isosurface image of left tibia.

**Fig. 3:**
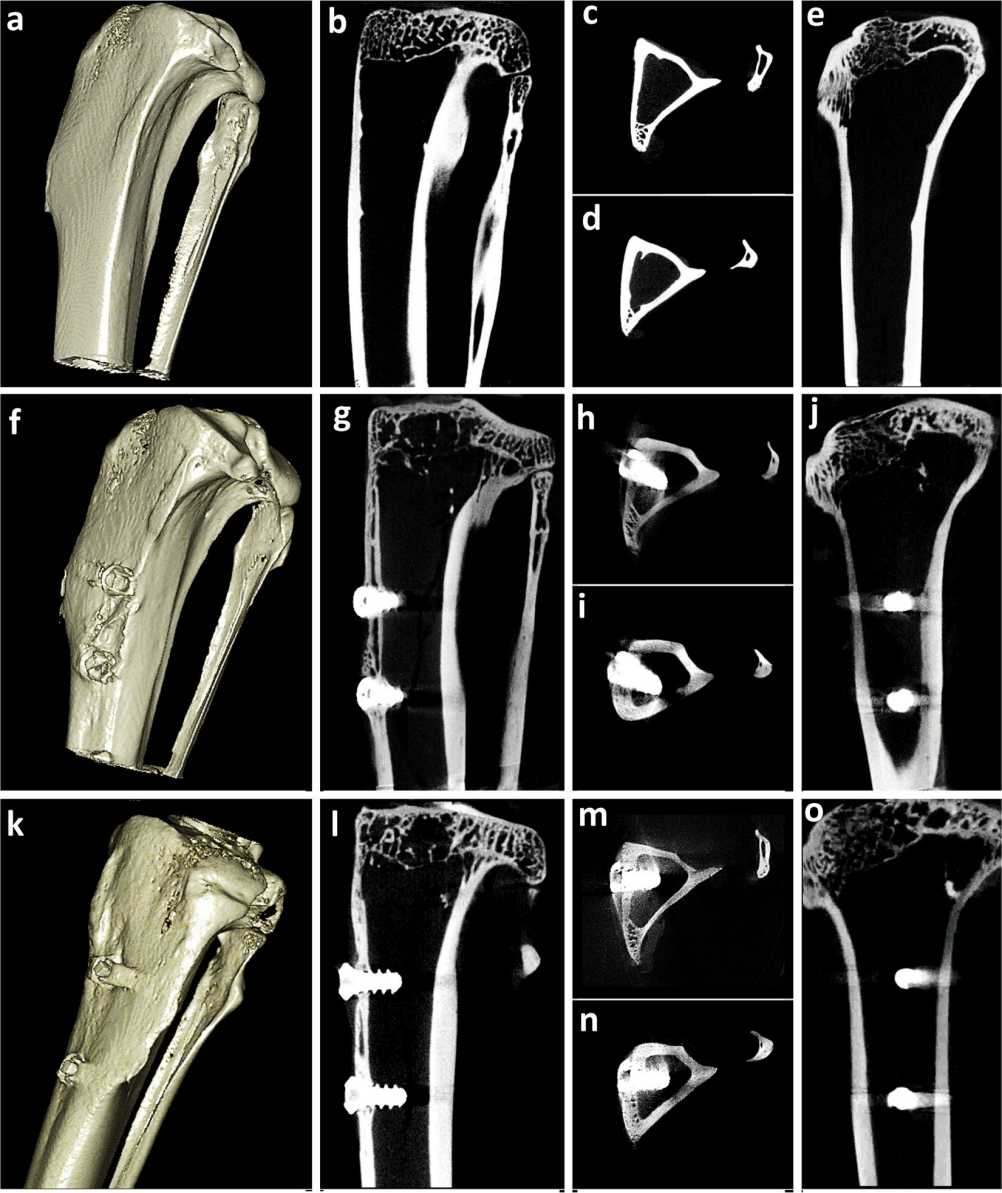
3D and 2D CT-scan of rabbit tibia. (**a**) 3D Volume rendering of rabbit tibia without implants (**b**–**e**) 2D sagittal, axial and coronal images, (**f**) 3D Volume rendering of tibia with test implants (**g**–**j**) 2D sagittal, axial and coronal images and (**k**) 3D Volume rendering of tibia with control implants (**i**–**o**) 2D sagittal, axial and coronal images respectively.

The manual segmentation method for calculating our defined parameter i.e. Implant bone integrated volume (IBIV) includes a constant rectangular contour over the peri-implant zone defining the area in the region of interest, located 1.3 mm proximally from the long axis of implant over two subsequent micro-CT slices (selected slices displayed the most pronounced and detail implant images in the entire series) for constituting volume of interest (VOI)^11,12^. A constant VOI was maintained with respect to size and shape for both test and control implants. A slice-wise 2D comparisons were performed for the defined peri-implant zone and original structure in order to secure exactness and genuine representation by the segmented region. Further to the discussion, a constant oval area is selected for the first and last slice for computing the volume for entire selected series of ROI in three planes (50 slices for axial/sagittal and 72 slices in coronal plane) (Fig. 2a–c). Briefly, the constant oval contours are manually outlined on the loaded images for test and control sample of 12^th^ week rabbit study model containing the implant and peri-implant tissue for evaluating 3D implant-bone volume^14^. Additionally, 3D reconstruction (Fig. 2d–h) (Fig. 3a–o) was performed as explained earlier using the algorithm of Feldkamp, Davis, and Kress^15^. Further, we choose an optimal threshold range that incorporates the gray values of the anatomical specimens for high-quality reconstructions (regions that are of no interest were not reconstructed). Subsequently, each reconstructed 3D models representing the precise anatomy of tibial implant bone complex were exported as a TIFF file from OsiriX software. It may be noted that although the current study is highly original, it is early and exploratory. The data sets for osteogenic nanofibrous coated implants were mainly acquired to demonstrate an example showing the potential possibilities, however with the provision that optimized scan parameters (i.e. voltage) are necessary to minimize the presence of possible artifacts, furthermore may be necessary to filter the obtained images.

## Data Records

The pre-clinical raw data sets along with DICOM files (n = 6 + 1) have been uploaded in  the Figshare repository^14^ that reveals the tomography slices of rabbit’s tibiae, were obtained at respective post-surgical study end points and have been used for segmentation of peri-implant regions followed by 3D reconstruction by a group of expert and experienced evaluators. As discussed earlier, the slice of tissue representing isotropic voxels are assigned a CT number as per x-ray attenuation properties of the respective tissue voxels. The major portion of the data consists of scanning micro-CT files of rabbit’s tibiae. The additional metadata consists of µCT‐derived density and bone morphometry measurements were also provided in .pdf files to represent the relevant information and current status of interfacial bone healing response in *metallo-biological* interface^14^. Here, the comparison of bone microarchitecture parameters of peri-implant tissue details regenerated with and without osteogenic nanofibrous coating for test and controls respectively were included in great detail apart from already accessible corroborating evidence derived from periotest values, pull out results and histomorphometry etc. that have been published earlier^12^.

**Fig. 4:**
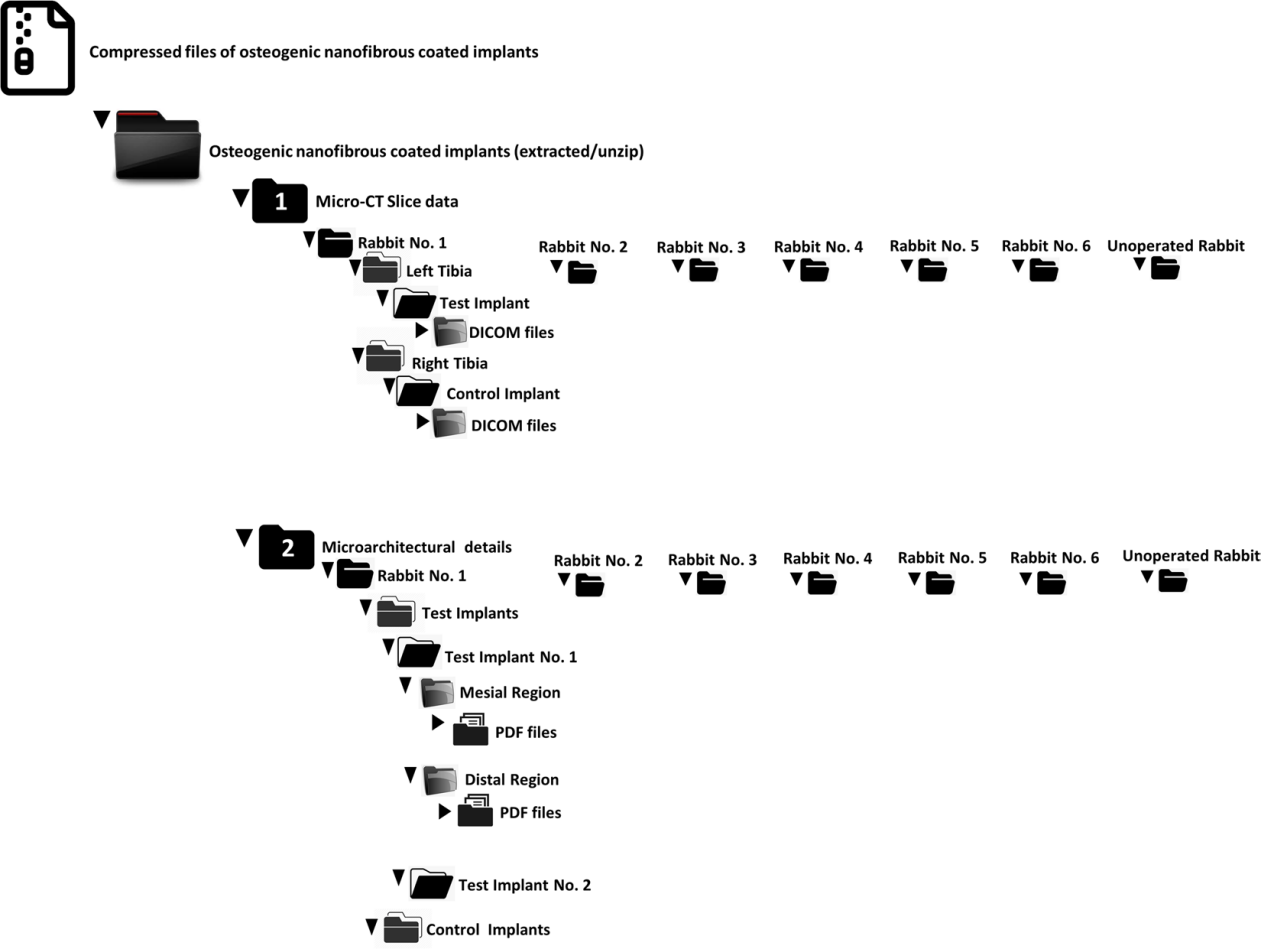
Data file organization in the data repository.

The dataset discussed and shared here incorporates all the rabbit’s data that were grouped and organized into 2 main folders namely micro-CT slice data and microarchitectural details (Table 2). Underneath the main folders, subfolders numbered through 1 to 6 (representing sequentially 6 rabbit study models) could be found (Table 2) (Fig. 4). Additionally, each subfolder under one of the main folder i.e. the folder of microarchitectural details were further divided into two subfolders containing test and control implants further leading to triplicate measurements of peri-implant mesial and distal regions of regenerated osseous tissues respectively (Table 2). We also wish to inform the prospective data users about a minor realignment in the form of implanting one additional titanium screw type implant in the left tibia of rabbit no.1 as the nanofibrous coating of one implant was not found to be sufficiently intact during surgical placement^11^. The datasets described here provides a precursory evidence in conjunction with other experimental details citing improved osseointegration of titanium implants when coated with osteogenic nanofibres^11,12^ thereby demonstrates the feasibility of our method and applicability^11,12,16^ towards possible consideration as a potential candidate for human clinical trials.

**Table 2:** Summary of data file arrangement in the data repository^14^.

Sl.No	File Name	Description (Triplicate measurements)	File format	File extension
1	**Osteogenic Nanofibrous Coated Implants**	Contains compressed Micro-CT DICOM and pdf files of all rabbits	Folder	.rar file
2	**Osteogenic Nanofibrous Coated Implants (Extracted)**	Contains Micro-CT DICOM and pdf files and folder of all rabbits	Folder	.dcm files, .pdf files
3	**Micro-CT Slice data**	Contains Micro-CT slices of all rabbit tibia in DICOM files	Folder	.dcm files
4	***Rabbit 1***	Contains subfolder of left and right tibia data for Rabbit 1 in DICOM files	Folder	.dcm files
5	Left Tibia	Contains Micro-CT data on left tibia in DICOM files	Folder	.dcm files
6	Test Implants	Contains Micro-CT data on left tibia with test implants in DICOM files	Folder contains terminal Micro-CT DICOM files of left tibia with test implants	.dcm files
7	Right Tibia	Contains Micro-CT data on right tibia in DICOM files	Folder	.dcm files
8	Control Implants	Contains Micro-CT data on right tibia with control implants in DICOM files	Folder contains terminal Micro-CT DICOM files of right tibia with control implants	.dcm files
	***Similar for Rabbit 2 to 6***	Contains subfolder of left and right tibia for Rabbits in DICOM files	Folder	.dcm files
34	***Similar for Unoperated Rabbit***	Contains subfolder of left and right tibia for unoperated rabbit in DICOM files	Folder	.dcm files
39	**Microarchitectural details**	Contains high‐resolution micro–computed tomography (µCT) data to evaluate peri-implant bone morphology	Folder	.pdf files
40	***Rabbit No. 1***	Contains Micro-CT bone microarchitectural details in subsequent subfolders	Folder	.pdf files
41	***Similar for Rabbit No. 2 to 6***	Contains Micro-CT bone microarchitectural details in subsequent subfolders	Folder	.pdf files
46	***Rabbit No. 7 with no implants***	Contains Micro-CT bone microarchitectural details in subsequent subfolders	Folder	.pdf files

## Technical Validation

The micro-CT imaging system discussed here was subjected to routine quality control protocols evaluating determinable quality control parameter available through images of manufacturers’ quality assurance wire and bone density phantoms. Additionally, the micro-CT scanning of tibial-implant-bone complex were performed with the predetermined sets of specification, generating a series of 2D projection data slices. The micro-CT system was based on optimized parameters i.e. in terms of voltages, current, exposure time etc. to name a few, for both test and control implants. Further, standardized operating protocol for scanning (e.g. projection number, rotation step etc.) and reconstruction (ring artefacts, metal artefacts, misalignment compensation, beam hardening corrections etc.) were ensured. Earlier, it has been claimed that even though histologic analyses provide comprehensive information regarding the cellular details and its dynamics, assessment of bone microarchitecture cannot be reliably ascertained^17^. Hence, further corroboration, reliability and effectiveness of our proof-of-concept micro-CT data were gauged by comparing macroscopic geometric details such as cortical plate thickness and dimensions of implants (in axial planes) between the tibial-implant-bone complex obtained from X-ray micro-CT images with that of traditional histological photomicrographs of the same specimen for all rabbit study models. The comparative data obtained from micro-CT and histological specimen reveals that the difference between the two techniques were not statistically significant leading to conclusion that the results obtained with micro-CT were similar to those obtained with histophotomicrograph images. Furthermore, all the measurements and comparison procedure were repeated in triplicate and were performed by experienced evaluators.

Scientific evidence shows that bone morphometry^18–20^ and preclinical volumetric imaging^21^ assessed by micro-CT has evolved gradually and revealed its crucial utility over the period of last few decades. Hence, with the addition of comparative micro-CT data of peri-implant tissues regenerated in response to our modified implant and controls to previously existing information of implant osseointegration and its dynamics; we have attempted to affirmatively endorse the advantages of our test implants via clarity and easy comparison between the alternative implants. Consequently, we have added the respective quantifiable details of bone microarchitecture to highlight the same. We envision that the present datasets will contribute positively to the developing information of implantology; specifically directed towards ideal implant-bone osseointegration.

## Usage Notes

For evaluating DICOM datasets described in the script^14^, image processing application such as Osirix® (https://www.osirix-viewer.com/) was utilized, however other 3D analysis software could also be used^22^. The 3D reconstructed structure of interests can be generated depending on the end user’s requirements using software packages mentioned above for morphometric and anatomic investigations and thereby assessing the validity of hypotheses.

## Data Availability

Peri-implant bone composition analyses were performed using MicroView 3D Image Viewer, an open source, dual-licensed, 3D image viewer (http://microview.sourceforge.net/) and OsiriX 8.5 software (https://www.osirix-viewer.com/) available commercially.
